# Infantile Kaposiform hemangioendothelioma in a female patient complicated with severe obstructed jaundice: a case report

**DOI:** 10.1186/s40792-022-01581-9

**Published:** 2022-12-29

**Authors:** Eiichiro Watanabe, Naoki Hashizume, Akihiro Yoneda, Mureo Kasahara, Genta Ozeki, Takeshi Saito, Michimasa Fujiogi, Motohiro Kano, Yuki Yamamoto, Osamu Miyazaki, Takanobu Maekawa, Noriyuki Nakano, Takako Yoshioka, Akihiro Fujino, Yutaka Kanamori

**Affiliations:** 1 Division of Surgery, Department of Surgical Specialties, National Center for Child Health and Development, Setagaya-Ku, Tokyo, Japan; 2 Division of Pediatric Surgical Oncology, National Center for Child Health and Development, Setagaya-Ku, Tokyo, Japan; 3 Organ Transplantation Center, National Center for Child Health and Development, Setagaya-Ku, Tokyo, Japan; 4 Department of Radiology, National Center for Child Health and Development, Setagaya-Ku, Tokyo, Japan; 5 Department of General Pediatrics and Interdisciplinary Medicine, National Center for Child Health and Development, Setagaya-Ku, Tokyo, Japan; 6 Department of Pathology, National Center for Child Health and Development, Setagaya-Ku, Tokyo, Japan

**Keywords:** Kaposiform hemangioendothelioma, Hepatoduodenal ligament, Sirolimus

## Abstract

**Background:**

Kaposiform hemangioendothelioma (KHE) is a rare locally aggressive vascular neoplasm that occurs mainly in the pediatric population. KHE usually originates just underneath the skin and affects deeper tissues through infiltrative growth; however, visceral tissue involvement is quite rare.

**Case presentation:**

An 8-month-old girl with jaundice and acholic stool was referred to our hospital for further evaluation of a hepatoduodenal ligament tumor. A blood examination revealed high bilirubin and liver enzyme levels, but no signs of coagulopathy. The first attempt at a diagnostic surgical procedure did not provide sufficient diagnostic information. However, the histopathological diagnosis of the cystic duct excised in the second surgery indicated KHE. Therefore, in our case, KHE was considered a cause of obstructive jaundice. Sirolimus (rapamycin) was initiated, and the patient was discharged 7 months after admission.

**Conclusions:**

In cases of atypical hypervascular lesions in the abdominal cavity, especially in the pediatric population, it is important to consider the possibility of KHE, and surgical intervention with proper strategies is required for diagnosis, followed sequentially by promising treatments.

## Background

Kaposiform hemangioendothelioma (KHE), first described in 1993 by Zukerberg [1], is a rare disease, locally aggressive vascular neoplasm that mainly occurs in the pediatric population [2]. KHE usually originates just beneath the skin and usually affects deeper tissues by infiltrative growth; however, visceral tissues involvement is quite rare. Several KHE cases with bone, retroperitoneal, or mediastinal involvement have been described [2]. We encountered a very rare case of infantile KHE complicated by obstructive jaundice. Herein, we present its difficult diagnosis.

## Case presentation

**Fig. 1:**
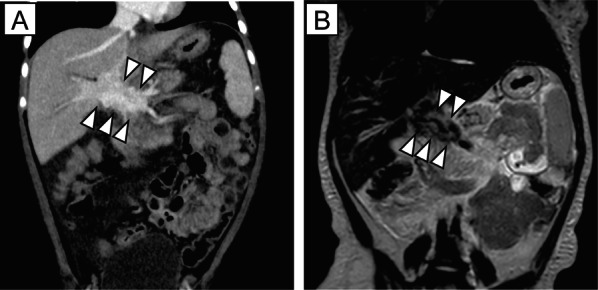
Contrast-enhanced computed tomography and magnetic resonance images obtained at admission. **A** Contrast-enhanced computed tomography (coronal reconstructed multiplanar image) revealing a large hypervascular tumor arising from the hepatoduodenal ligament (HDL) (arrowheads). **B** Coronal T2-weighted image showing a mass-like lesion with a low-intensity area along the HDL

An 8-month-old female patient with jaundice and acholic stool was taken to a hospital, where blood examinations revealed elevated liver enzyme (aspartate transaminase/alanine transaminase [AST/ALT], 254/161 U/L) and bilirubin (total, 5.6 mg/dL) levels without signs of coagulopathy. Abdominal computed tomography and magnetic resonance imaging showed a large hypervascular tumor arising from the hepatoduodenal ligament (HDL) (Fig. 1). She was referred to our hospital with obstructive jaundice due to a tumor originating from the HDL.

To diagnose the tumor, we performed an exploratory laparotomy on hospital day 2. No tumor-like lesions were observed in the upper abdominal cavity. The HDL became edematous and hard and bled easily, indicating acute inflammation. En bloc surgical excision of the lesion is considered associated with a risk of injury to the bile duct, hepatic artery, and portal vein. Therefore, a liver biopsy and sampling of the HDL and lymphoid tissue around the neck of the gall bladder were performed. However, these surgical procedures did not provide a definitive diagnosis.

Despite the initiation of oral corticosteroid treatment, the jaundice did not subside (total/direct bilirubin, 9.1/6.6 mg/dL), and the AST/ALT levels were elevated (322/260 U/L). The dilatation of the intrahepatic bile duct gradually became exacerbated, and the gallbladder became quite large and palpable over the surface of the abdominal wall. Spontaneous rupture of the gallbladder is a cause for concern. Therefore, on postoperative day 32, the patient underwent a biopsy of the lesion and a cholecystectomy.

**Fig. 2:**
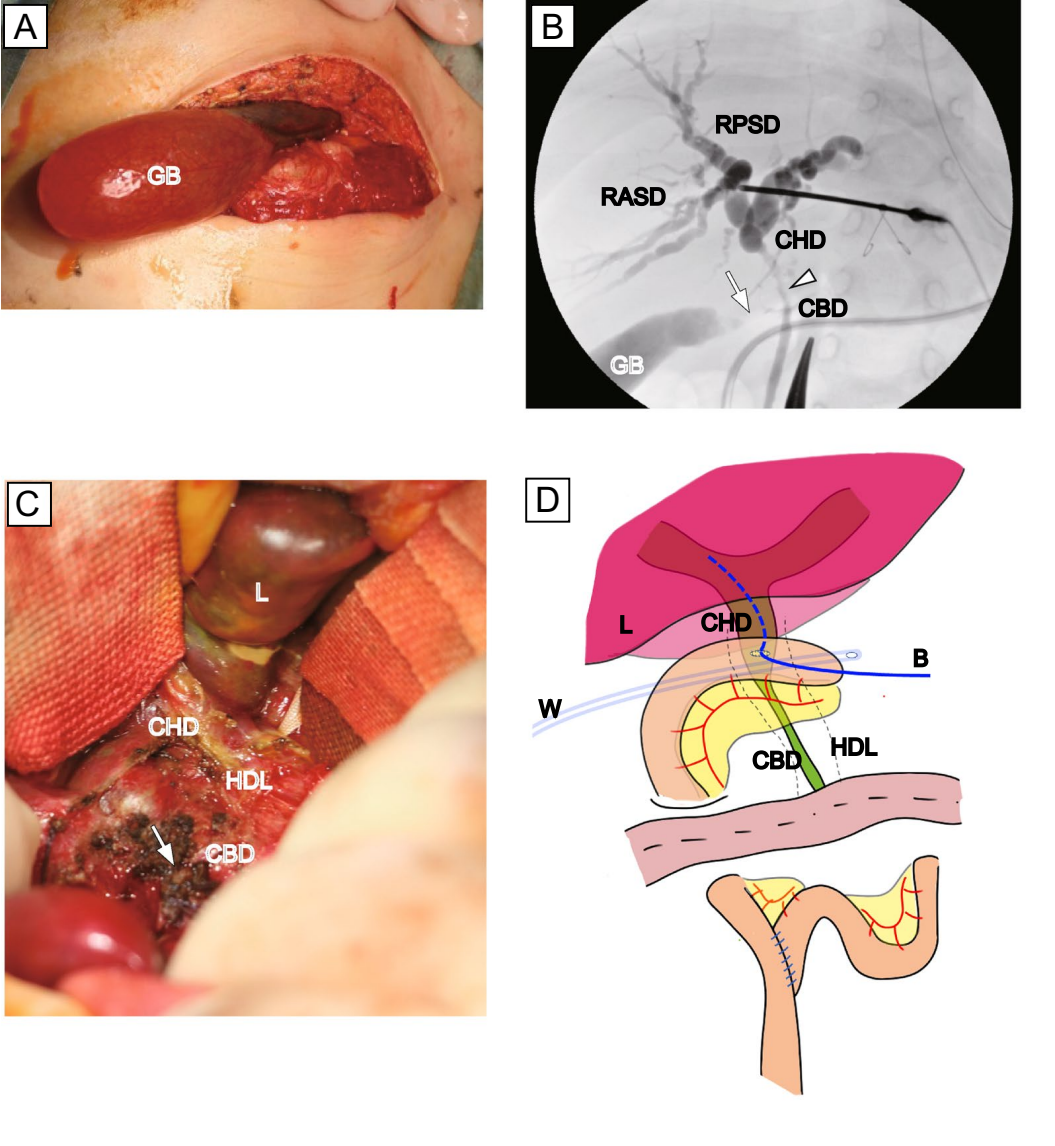
Intraoperative findings of second operation.** A** The GB appears quite distended with white bile. **B** Intraoperative cholecystography revealed that the CD is completely obstructed (arrow), and intraoperative intrahepatic cholangiography revealed narrowing of the bile duct around the junction of the CD along the hepatoduodenal ligament (arrowhead). CBD, common bile duct; CD, cystic duct; CHD, common hepatic duct; GB, gallbladder; RASD, right anterior segment duct; RPSD, right posterior segment duct. **C** Area around the junction of the CD and CHD. The CD was resected as distally as possible (arrow). CD, cystic duct; CHD, common hepatic duct; HDL, hepatoduodenal ligament. **D** Hepaticojejunostomy performed in Roux-en-Y fashion. The jejunum was anastomosed with the ventral wall of the CHD. A 4-Fr hepatic drainage tube was placed in Witzel fashion (B) and a 15-Fr drainage tube was inserted via the Winslow foramen (W)

The HDL became soft and less edematous but bled easily, indicating subacute inflammation. Despite a detailed observation, no obvious lesions were observed in the abdominal cavity. Intraoperative cholecystography revealed that the cystic duct (CD) was completely obstructed, and intraoperative intrahepatic cholangiography revealed that the bile duct around the junction of the CD narrowed along the HDL. Obvious lesions were difficult to obtain; therefore, a cholecystectomy with CD resection as distally as possible, liver biopsy, and no. 12b lymphoid tissue sampling were performed. To improve the severe obstructive jaundice, a hepaticojejunostomy in a Roux-en-Y fashion was performed in which the jejunum was anastomosed to the ventral wall of the common hepatic duct (CHD) (Fig. 2). Almost all the lesions remained in the second surgery.

### Histopathological findings

**Fig. 3:**
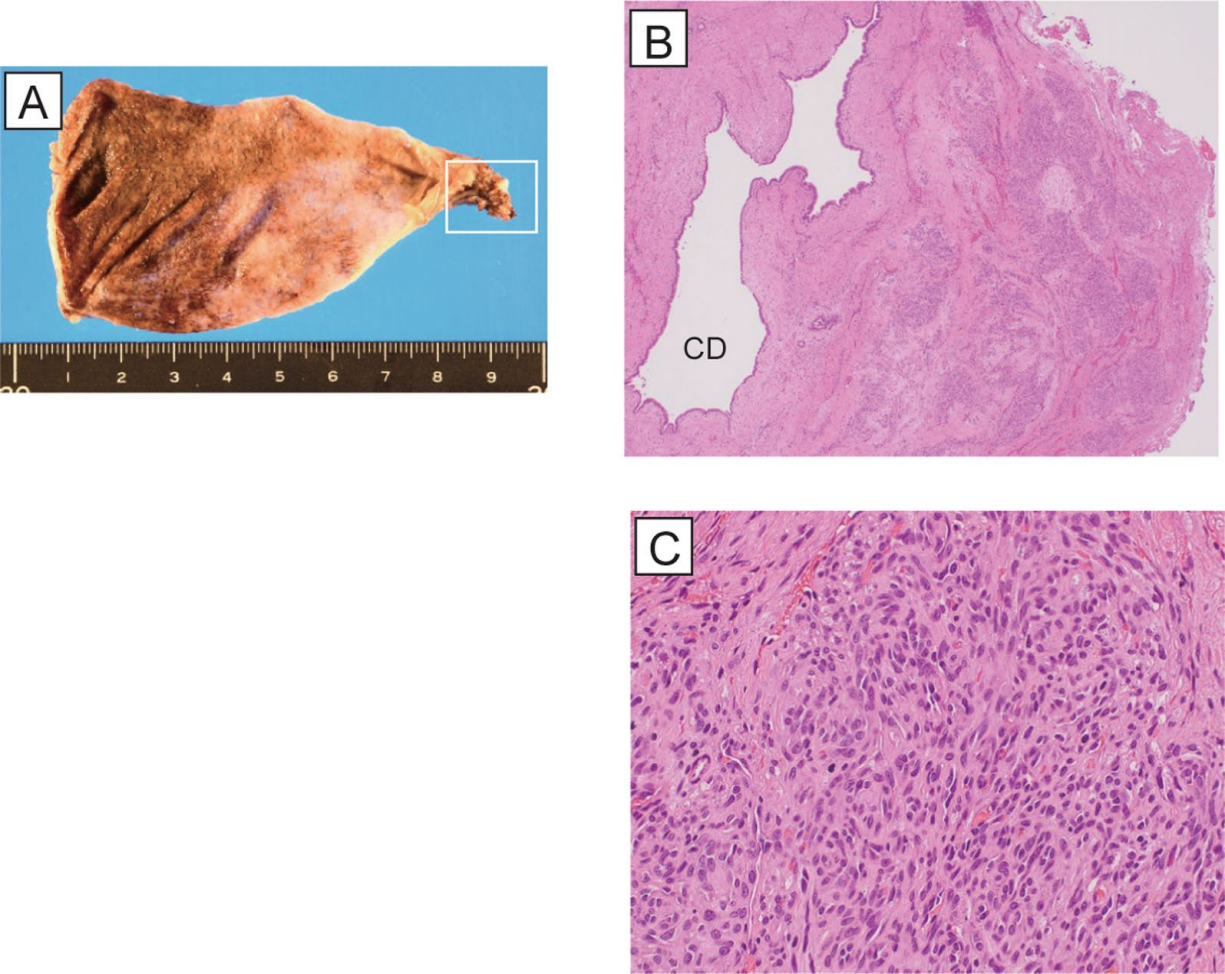
Histopathological findings of the cystic duct.** A** Macroscopic findings of the gallbladder. The cystic duct positioned close to the hepatoduodenal ligament is a clue to the diagnosis of Kaposiform hemangioendothelioma (white line square). **B** H&E stained section of the peri-cystic duct. Hypervascular and polynodular lesions were observed around the resected CD (× 40). CD, cystic duct. **C** H&E stained section of the peri-cystic duct. Proliferated spindle cells formed slit-like lumens filled with red blood cells (× 400)

Hypervascular and polynodular lesions were observed around the resected CD close to the HDL in which proliferating spindle cells formed slit-like lumens filled with red blood cells. Immunohistochemical staining demonstrated that the spindle cells were positive for CD31, CD34, and ERG, partially positive for D2-40 and Prox1, and negative for GLUT1. These findings are consistent with those of KHE (Fig. 3).

### Postoperative clinical course

**Fig. 4:**
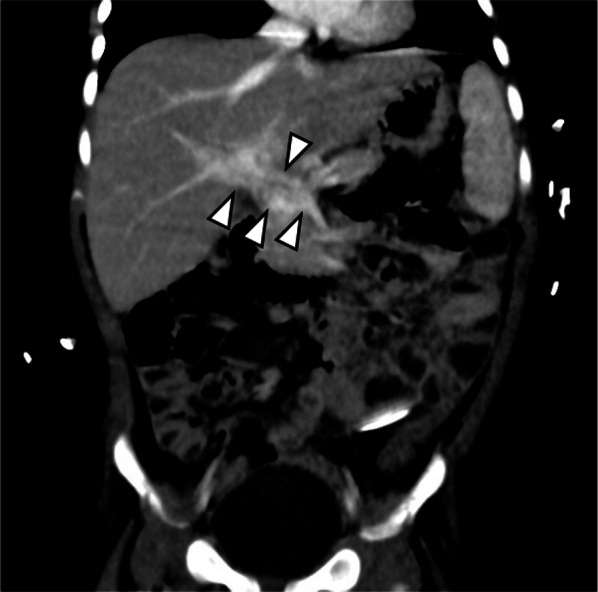
Contrast-enhanced computed tomography obtained at 87 days postoperative. Contrast-enhanced computed tomography (coronal reconstructed multiplanar image) revealed that a hypervascular tumor arising from the hepatoduodenal ligament was slightly reduced (arrowheads) after sirolimus (rapamycin) was started

The patient’s obstructive jaundice improved rapidly after the hepaticojejunostomy during the second surgical intervention. In contrast, the amount of ascites was significantly increased in the early postoperative period, and the ascites were presumed to comprise chyle and lymph fluids. With symptomatic therapy, the oral administration of 0.5 mg/day (0.06 mg/kg/day) sirolimus (rapamycin) was started as a first-line therapy on postoperative day 24 (trial registration jRCTs031180290). The large amount of ascites gradually decreased, and the hepatic and Winslow drainage tubes were removed at 83 and 90 days postoperative, respectively. The KHE lesion was slightly reduced (Fig. 4). The patient was discharged 7 months after admission.

## Discussion

KHE is a rare locally aggressive vascular neoplasm that occurs mainly in the pediatric population [2]. KHE usually originates just beneath the skin and affects deeper tissues through infiltrative growth; however, visceral tissue involvement is quite rare. Although KHE may involve various organs such as bone, retroperitoneum, and mediastinum, involvement of the choledochus is rare [2, 3].

To the best of our knowledge, five cases of KHE originating from the HDL area have been reported in English (Table 1). Terui et al. reported the case of a 5-month-old boy who was successfully treated with hepatoportoenterostomy [3]. In contrast, Mathew et al. reported a demised case of an 8-month-old female infant [4]. In addition, Yao et al. reported three clinically diagnosed cases in male patients with obstructive jaundice and Kasabach–Merritt phenomenon (KMP), all of whom were successfully diagnosed without surgical intervention and administered sirolimus (rapamycin) as primary treatment. Their clinical course was satisfactory with no additional surgical interventions [5, 6].

**Table 1:** Summary of clinical characteristics of infantile KHE

Case	Author	Year	Age	Sex	Origine	KMP	Diagnosis	Drainage	SI	Medication	Prognosis
1	Terui [3]	2010	5 m	M	CBD	(−)	SI	(−)	HJ	(−)	Alive
2	Mathew [4]	2019	4 m	F	HP	(+)	SI	(−)	(−)	IFN	Loss
3	Yao [5]	2020	50 d	M	PH/HP	(+)	I/CS	PTCD	(−)	S/VCR/SLR	Alive
4			80 d	M	PH/HP	(+)	I/CS	PTBD	(−)	S/VCR/SLR	Alive
5			73 d	M	HP	(+)	I/CS	(−)	(−)	S/VCR/SLR	Alive
6	Present case	2022	8 m	F	HDL	(−)	SI	(−)	HJ	S/SLR	Alive

*CBD* common bile duct, *CS* clinical symptoms, *HDL* hepatoduodenal ligament, *HJ* hepaticojejunostomy, *HP* head of the pancreas, *I* images, *IFN* interferon, *KHE* Kaposiform hemangioendothelioma, *KMP* Kasabach–Merritt phenomenon, *PH* porta hepatis, *PTBD* percutaneous transhepatic biliary drainage, *PTCD* percutaneous transhepatic cholangiodrainage, *SI* surgical intervention, *SLR* sirolimus, *VCR* vincristine

In most cases, KHE is associated with consumptive KMP or lymphangiomatosis [2], and these clinical symptoms could be diagnostic clues that lead to the diagnosis of KHE, even in atypical cases [5]. In our case, KHE was not considered until the histopathological diagnosis was demonstrated because our case did not show coagulopathy or distinct lymphangiomatosis. However, in cases of atypical visceral tissue involvement without typical clinical symptoms, it is important to consider KHE in the differential diagnosis.

Regarding the treatment of KHE, definitive treatment guidelines are unavailable due to the very low incidence of this disease. Corticosteroids are often used as first-line therapy; however, they feature some adverse effects such as hypertension, a cushingoid appearance, growth retardation, and opportunistic infections, and they are infrequently effective as solitary agents. Wang et al. suggested vincristine (VCR), an inhibitor of endothelial proliferation, as a first-line treatment for steroid-resistant cases [7]. Sirolimus (rapamycin), an mTOR pathway inhibitor, was recently identified as beneficial for patients with KHE and VCR-resistant and -refractory or recurrent life-threatening KMP [5]. In our case, silorimus (rapamycin) was administered after KHE was diagnosed, and the clinical symptoms, such as postoperative ascites, gradually improved. Therefore, the oral administration of sirolimus (rapamycin) is considered effective for complicated KHE originating from HDL.

In addition, surgical excision could be an effective therapeutic option; however, this is not always possible due to the disease location, infiltrative pattern of these tumors, and presence of thrombocytopenic coagulopathies [7]. In our case, liver transplantation (LT) was considered for surgical excision of the tumor; however, the liver was not cirrhotic; thus, LT was considered the last therapeutic option.

For the rescue of the obstructive jaundice, surgical interventions such as percutaneous transhepatic cholangiodrainage (PTCD) and hepaticojejunostomy were considered. If PTCD was selected instead of hepaticojejunostomy, some troubles such as infection and obstruction of the drainage tube were encountered in the long course of hospitalization. In our case, a hepaticojejunostomy effectively reduced the obstructive jaundice. The jejunum was anastomosed to the ventral wall of the CHD because the tumor infiltrated the HDL and it was quite challenging to expose the CHD without intraoperative complications. However, our modification was reflected in the postoperative course, and CD resection as distally as possible was a definitive factor for the diagnosis. Therefore, if obstructive jaundice is caused by a tumor of unknown origin inside the HDL, these challenges could become diagnostic clues followed by definitive treatment.

## Conclusion

We encountered a very rare case of intra-abdominal KHE complicated by severe obstructive jaundice. Unless there are no signs of KMP or lymphangiomatosis, in cases of atypical hypervascular lesions in the abdominal cavity, especially among infants, it is important to consider the possibility of KHE, and surgical intervention with proper strategies is required for the diagnosis, followed by promising treatment.

## Data Availability

Not applicable.

## Abbreviations

AST: Asparate aminotransferase
ALT: Alanine aminotransferase
CD: Cystic duct
CHD: Common hepatic duct
HDL: Hepatoduodenal ligament
HE: Hemangioendothelioma
KHE: Kaposiform hemangioendothelioma
KMP: Kasabach–Merritt phenomenon
LT: Liver transplantation
PTBD: Percutaneous transhepatic biliary drainage
VCR: Vincristine

## References

[1] Zukerberg LR, Nickoloff BJ, Weiss SW (1993). Kaposiform hemangioendothelioma of infancy and childhood. An aggressive neoplasm associated with Kasabach-Merritt syndrome and lymphangiomatosis. Am J Surg Pathol.

[2] Fernandez Y, Bernabeu-Wittel M, Garcia-Morillo JS (2009). Kaposiform hemangioendothelioma. Eur J Intern Med.

[3] Terui K, Nakatani Y, Kambe M, Fukunaga M, Hishiki T, Saito T (2010). Kaposiform hemangioendothelioma of the choledochus. J Pediatr Surg.

[4] Mathew D, Mahomed N (2019). Pancreatic kaposiform hemangioendothelioma complicated by Kasabach-Merritt phenomenon: a rare entity. SA J Radiol.

[5] Yao W, Li K, Wang Z, Dong K, Zheng S (2020). Retroperitoneal kaposiform hemangioendothelioma complicated by Kasabach-Merritt phenomenon and obstructive jaundice: a retrospective series of 3 patients treated with sirolimus. Pediatr Dermatol.

[6] Hase T, Kodama M, Kishida A, Matsushita M, Kurumi Y, Mizukuro T (1995). Successful management of infantile hepatic hilar hemangioendothelioma with obstructive jaundice and consumption coagulopathy. J Pediatr Surg.

[7] Wang Z, Li K, Yao W, Dong K, Xiao X, Zheng S (2015). Steroid-resistant kaposiform hemangioendothelioma: a retrospective study of 37 patients treated with vincristine and long-term follow-up. Pediatr Blood Cancer.

